# Circular RNA CpG island hypermethylation-associated silencing in human cancer

**DOI:** 10.18632/oncotarget.25673

**Published:** 2018-06-26

**Authors:** Humberto J. Ferreira, Veronica Davalos, Manuel Castro de Moura, Marta Soler, Montserrat Perez-Salvia, Alberto Bueno-Costa, Fernando Setien, Sebastian Moran, Alberto Villanueva, Manel Esteller

**Affiliations:** ^1^ Cancer Epigenetics and Biology Program (PEBC), Bellvitge Biomedical Research Institute (IDIBELL), Barcelona, Catalonia, Spain; ^2^ Laboratory of Translational Research, Catalan Institute of Oncology (ICO), Bellvitge Biomedical Research Institute (IDIBELL), Barcelona, Catalonia, Spain; ^3^ Centro de Investigacion Biomedica en Red Cancer (CIBERONC), Madrid, Spain; ^4^ Physiological Sciences Department, School of Medicine and Health Sciences, University of Barcelona (UB), Barcelona, Catalonia, Spain; ^5^ Institucio Catalana de Recerca i Estudis Avançats (ICREA), Barcelona, Catalonia, Spain

**Keywords:** circular RNA, noncoding RNA, DNA methylation, cancer, epigenetics

## Abstract

Noncoding RNAs (ncRNAs), such as microRNAs and long noncoding RNAs (lncRNAs), participate in cellular transformation. Work done in the last decade has also demonstrated that ncRNAs with growth-inhibitory functions can undergo promoter CpG island hypermethylation-associated silencing in tumorigenesis. Herein, we wondered whether circular RNAs (circRNAs), a type of RNA transcripts lacking 5′-3′ ends and forming closed loops that are gaining relevance in cancer biology, are also a target of epigenetic inactivation in tumors. To tackle this issue, we have used cancer cells genetically deficient for the DNA methyltransferase enzymes in conjuction with circRNA expression microarrays. We have found that the loss of DNA methylation provokes a release of circRNA silencing. In particular, we have identified that promoter CpG island hypermethylation of the genes TUSC3 (tumor suppressor candidate 3), POMT1 (protein O-mannosyltransferase 1), ATRNL1 (attractin-like 1) and SAMD4A (sterile alpha motif domain containing 4A) is linked to the transcriptional downregulation of both linear mRNA and the hosted circRNA. Although some circRNAs regulate the linear transcript, we did not observe changes in TUSC3 mRNA levels upon TUSC3 circ104557 overexpression. Interestingly, we found circRNA-mediated regulation of target miRNAs and an *in vivo* growth inhibitory effect upon TUSC3 circ104557 transduction. Data mining for 5′-end CpG island methylation of TUSC3, ATRNL1, POMT1 and SAMD4A in cancer cell lines and primary tumors showed that the epigenetic defect was commonly observed among different tumor types in association with the diminished expression of the corresponding transcript. Our findings support a role for circRNA DNA methylation-associated loss in human cancer.

## INTRODUCTION

Approximately just 2% of the genome is transcribed into protein-coding RNAs [1], so the majority of transcripts are noncoding RNAs (ncRNAs), that can be categorized according to their structural properties and length [2]. Among the small ncRNAs, the most well-studied class are microRNAs (19–25 nt) that control gene expression through complementarity with target mRNAs [3]. In human tumors, miRNA expression patterns are distinct between normal tissues and derived malignancies, and between different tumor types [4, 5]. miRNAs can act as tumor suppressors or oncogenes, having a central role in oncogenesis [6]. Related to miRNAs showing growth inhibitory functions, their defects are associated with diverse mechanisms such as an impairment miRNA post-transcriptional regulation, targeted repression by oncogenes and the hypermethylation of the CpG island promoter located in the corresponding genomic locus from where the miRNA is transcribed [7, 8]. Long ncRNAs (lncRNAs; arbitrarily >200 nt) constitute another class of ncRNAs that, despite the lack of protein-coding potential, commonly display mRNA-like features, such as poly(A) tails and multiexonic gene structures [2, 9]. LncRNAs are linked with a variety of functions, including splicing regulation, chromatin-related functions, and transcriptional control [2, 9]. Aberrant expression of lncRNAs in human tumors is common, and some lncRNAs have been demonstrated to act as oncogenes or tumor suppressors [2, 10]. The altered lncRNA expression of cancer cells can be associated with genetic events, such as copy number changes, but in an increasing number of cases, lncRNAs are targets of epigenetic silencing. In this regard, transcribed–ultraconserved regions [11, 12], small nucleolar RNAs [13], antisense RNAs [14] and other types of lncRNAs [15], have been demonstrated to undergo cancer-specific promoter CpG island hypermethylation-associated loss.

Another novel and increasingly important class of noncodingRNAs are circular RNAs (circRNAs). CircRNAs are transcripts that lack 5′-3′ ends and poly(A) tails, forming a covalent closed loop [16, 17]. CircRNAs were first originally discovered in viruses in the 1970s [18], although they were mainly considered as transcriptional noise or splice errors. The recent re-discovery of circRNAs has been possible by the generalization of RNA sequencing methodology, particularly those that are not based on poly(A) purification, as well as new bioinformatic and quantitative PCR tools. Although some artificial circRNAs with an internal ribosome entry site (IRE) have been translated *in vitro* and a few endogenous circRNAs have been able to generate proteins, the evidence for their translation in the natural setting is limited [19] and most circRNAs are considered as ncRNAs. In this setting, the functions of circRNAs are diverse, with a proposed predominat role as miRNA sponges or decoys in association with their most common location in the cytoplasm, as well as exerting roles in protein scaffolding and in the regulation of RNA splicing and transcription [16, 17]. Most importantly, circRNA expression profiles are disrupted in many tumor types in comparison with their normal tissue counterparts [17, 20]. In this regard, some circRNAs have been characterized as pro-oncogenic, such as ciRS-7-A that enhances the EGFR/RAF1/MAPK pathway [21], or tumor suppressor molecules, such as circFoxo3 that prevent MDM2 activities [22].

Despite the growing evidence of the role of circRNAs in tumorigenesis, we know very little about how their expression and activity become disrupted in cancer cells. CircRNAs are thought to be mainly derived from the processing of precursor RNA (pre-mRNA) backsplicing of exons. A common scenario would be that a downstream 5′ splice site (donor) of an exon joins an upstream 3′ splice cite (acceptor) to originate the circRNA [16, 17]. Upstream defects in genes involved in pairing of complementary sequences and, thus, probably in backsplicing efficiency, can affect circRNA levels. This would be the case for the DexH-box helicase 9 (DHX9), adenosine deaminase acting on RNA-1 (ADAR1), fused in sarcoma (FUS), heterogeneous nuclear ribonucleoprotein (hnrNP) or Quaking (QKI) [17, 20]. Genomic aberrations can also contribute to abnormal circRNA activities, as it has been demonstrated by the identification of translocations that generate fusion circRNAs (f-circRNAs) that contribute to cellular transformation [23]. However, considering that many circRNAs are downregulated in cancer [24], and both tumor suppressor protein-coding genes [25] and the ncRNAs described above undergo DNA methylation-associated silencing in tumors, we wondered if circRNAs are also targeted by this type of epigenetic inactivation. Herein, using genomic approaches that determine circRNA expression profiles vs DNA methylation patterns, we provide evidence that circRNAs, in conjuction with their corresponding linear RNAs, undergo cancer-specific hypermethylation-associated transcriptional silencing. These findings establish the epigenetic loss of circRNAs as another common characteristic of human tumors.

## RESULTS

### Cancer-specific promoter CpG island hypermethylation silences circular and host linear RNA

**Figure 1:**
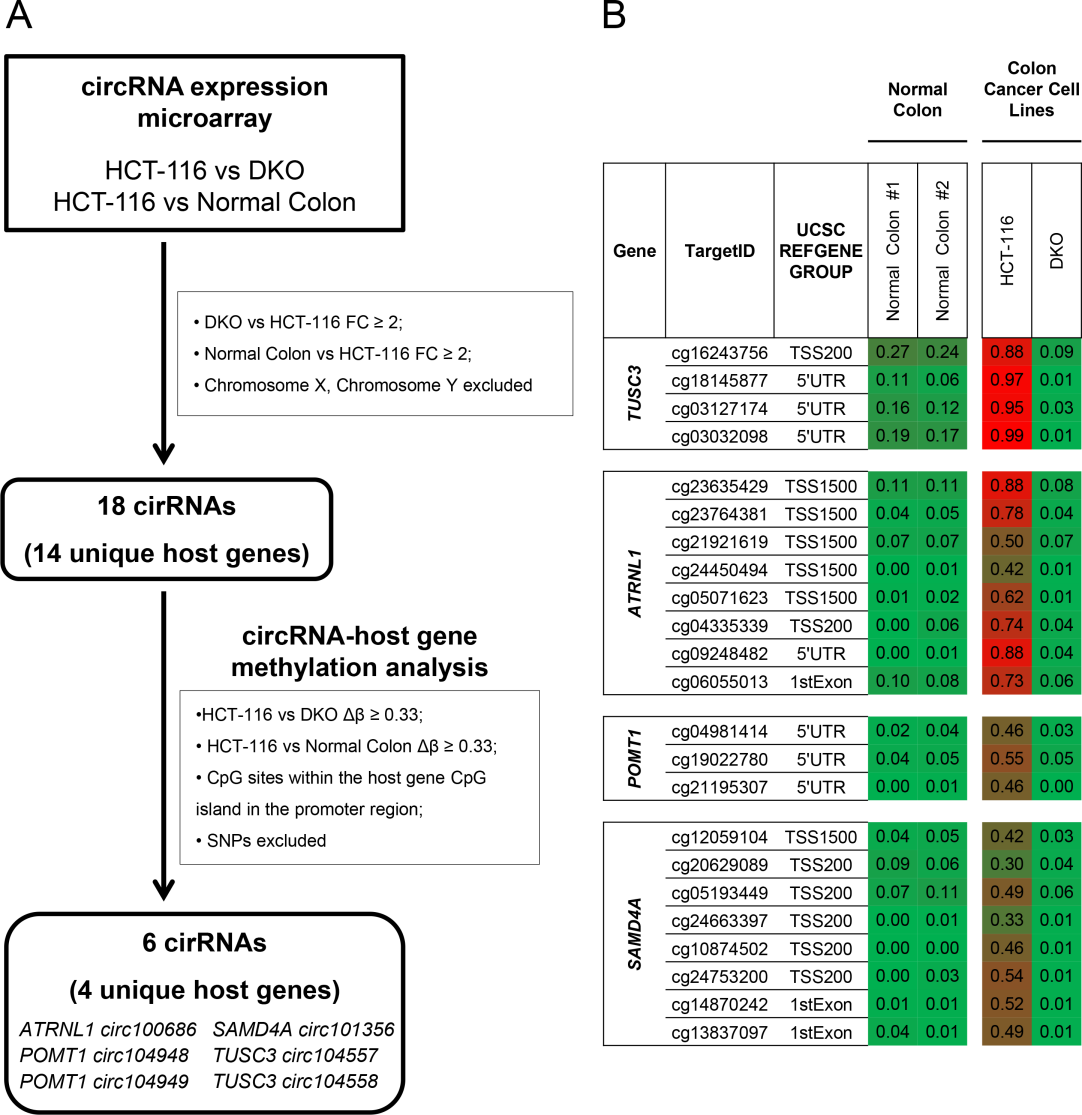
Screening for CpG island hypermethylation-associated silencing of circular RNAs in cancer cells (**A**) Flow-chart used to identify candidate circRNAs silenced in colon cancer through CpG island hypermethylation in the promoter region of their host genes. FC, Fold Change; SNP, Single Nucleotide Polymorphism. (**B**) DNA methylation profile of the 5′-end CpG island regulatory region for the TUSC3, ATRNL1, POMT1 or SAMD4A genes analyzed by the 450K DNA methylation microarray. DNA methylation data for healthy colon mucosa correspond to two normal colon patient samples available at TCGA (Normal colon 1: TCGA-A6-2675-11A, sigmoid colon normal tissue; Normal colon 2: TCGA-A6-2685-11A, sigmoid colon normal tissue). Single CpG absolute methylation levels (0–1) are shown. Green, unmethylated; red, methylated. Data from normal colon, HCT-116 and DKO cells are shown.

To identify cancer-specific candidate DNA methylation changes that affect circRNA expression, we used the experimental workflow displayed in Figure 1A. We took advantage of the development of an isogenic cell line obtained from the human colon cancer cell line HCT-116, where the DNA methyltransferases 1 (DNMT1) and 3B (DNMT3B) genes have been genetically disrupted by homologous recombination (double knockout cells, DKO) [26]. DKO cells show highly reduced DNMT activity, 5-methylcytosine DNA levels and release of coding genes and lncRNA silencing associated with promoter CpG island hypomethylation [15, 26]. Supplementary Figure 1 shows by semiquantitative RT-PCR of mRNA and western blot that our DKO cells are deficient in the mentioned DNA methyltransferase enzymes. We sought to characterize tumor-specific DNA methylation events in circRNA loci, so we also added a sample of normal colon mucosa in our screening strategy. To assess circRNA levels, total RNA from HCT-116, DKO and normal colon mucosa was analyzed using a previously validated circRNA microarray platform [27, 28]. Using this circRNA expression microarray, we interrogated a total of 4,998 human circRNAs. The circRNA microarray expression data obtained are available at the GEO repository under number GSE109676. According to their origins, these circRNAs can be classified as 4,637 (92.7%) “Exonic” (arising from the exons of the linear transcript), 233 (4.7%) “Intronic” (arising from an intron of the linear transcript), 75 (1.5%) “Intragenic” (representing circRNAs transcribed from same gene locus as the linear transcript, but not classified into “exonic” and “intronic”), 52 (1.04%) “Antisense” (representing circRNAs whose gene loci overlap with the linear RNA, but that are transcribed from the opposite strand) and only one (0.02%) “Intergenic” (representing a circRNA located outside of any known gene loci). Using this expression platform, we identified 18 circRNAs (0.36% of the total 4,998) that were not expressed in the colorectal cancer cell line HCT-116, but whose expression was restored in DKO cells and that were also expressed in normal colorectal mucosa. None of the circRNAs expressed in normal colorectal mucosa was overexpressed in HCT-116 compared to DKO cells. All the DKO upregulated circRNAs were classified as “exonic” circRNAs included in 14 unique host genes. These circRNAs were FAM190B hsa_circRNA_100637, FAM190B hsa_circRNA_100638, FBXO9 hsa_circRNA_104122, GTF2IRD2 hsa_circRNA_104402, IFT46 hsa_circRNA_100959, NEK11 hsa_circRNA_103474, SGMS1 hsa_circRNA_100598, SLC37A3 hsa_circRNA_104510, ZNF532 hsa_circRNA_102379, ACVR2A circ102830, TTBK2 circ101496, TTBK2 circ101498, ATRNL1 circ100686, POMT1 circ104948, POMT1 circ104949, SAMD4A circ101356, TUSC3 circ104558 and TUSC3 circ104557.

To characterize among these circRNAs those with tumor-specific differential promoter CpG island methylation of their respective host genes, we analyzed the DNA methylation profile of HCT-116 cells, the derived DKO cells, and normal colon. DNA methylation patterns were determined using the Infinium HumanMethylation450 (450K) microarray, which includes 482,422 CpG sites [29]. We observed that among the 14 host genes that originated the 18 circRNAs identified above, the 5′-end CpG islands of the four protein coding genes TUSC3 (hosting TUSC3 circ104558 and TUSC3 circ104557), ATRNL1 (hosting ATRNL1 circ100686), POMT1 (hosting POMT1 circ104948 and POMT1 circ104949) and SAMD4A (hosting SAMD4A circ101356) were hypermethylated in HCT-116 cells and unmethylated in DKO cells and normal colon mucosa (Figure 1B). Thus, these four genes and their six hosted circRNAs became our main candidates to undergo cancer-specific DNA methylation silencing and were studied in more detail.

**Figure 2:**
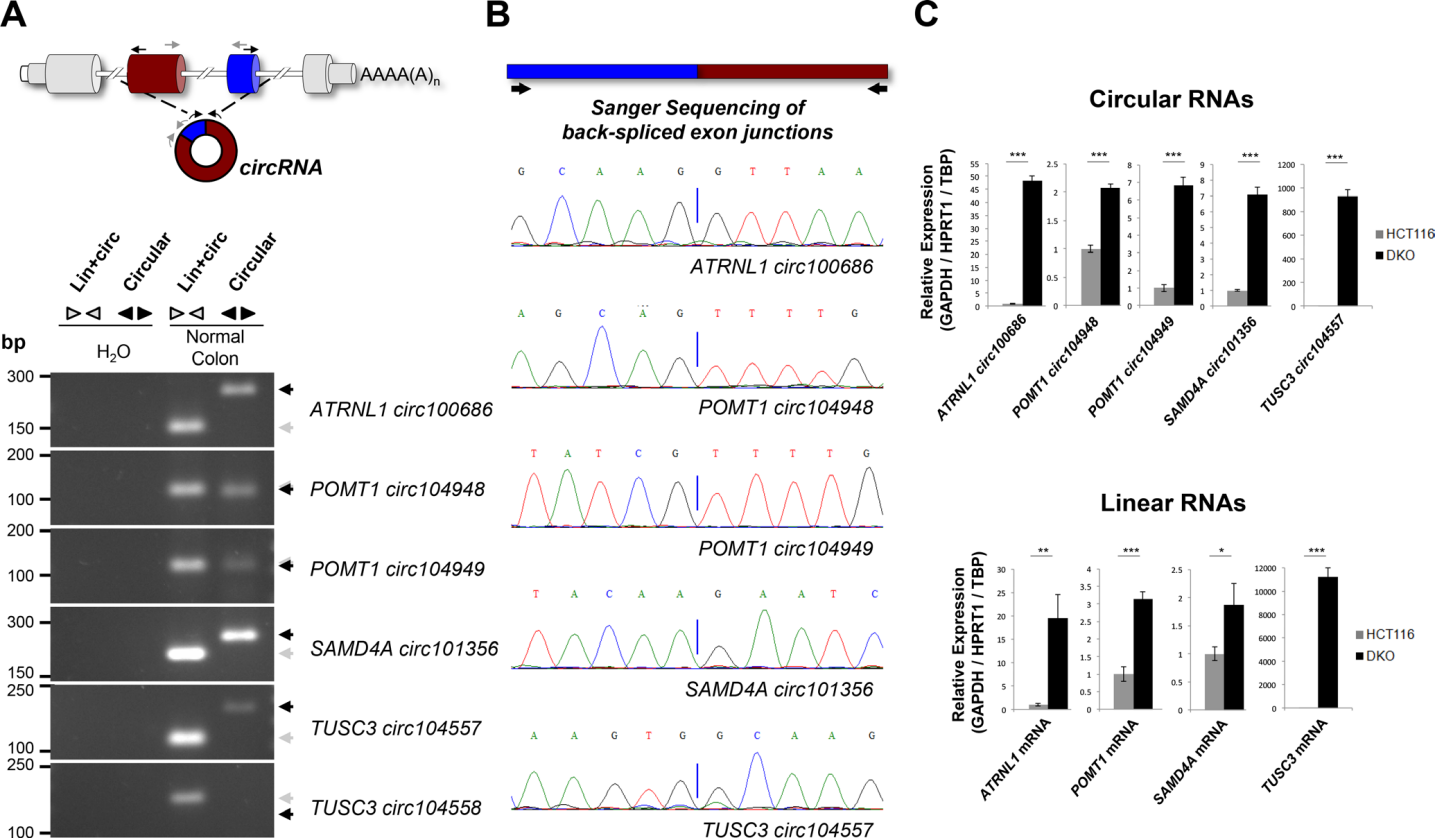
Characterization of TUSC3, ATRNL1, POMT1 and SAMD4A promoter CpG island hypermethylation-associated silencing of circular and linear RNAs (**A**) Convergent and divergent PCRs in normal colon, amplifying both circular and linear transcripts, or only the circular transcript, respectively. Since circRNAs encoded within POMT1 host gene shared a common sequence, convergent primers were designed for that region. Five out of six circRNAs were amplified in normal colon: ATRNL1 circ100686, POMT1 circ104948, POMT1 circ104949, SAMD4A circ101356 and TUSC3 circ104557. The TUSC3 circ104558 was not detected and it was excluded from the study. (**B**) Sanger Sequencing was used to sequence the head-to-tail junctions present in the fragments amplified by divergent PCR amplification. Chromatograms confirmed the occurrence of back-splicing. (**C**) Real-time quantitative PCR of both circular (*top*) and linear (*below*) transcripts in HCT-116 and DKO cells. Primers designed to amplify linear transcripts do not overlap annotated circRNAs according to circBase. Expression levels are relative to three housekeeping genes (GAPDH/HPRT1/TBP). Error bars, SD from three biological replicates. ^*^p ≤ 0.05, ^**^p ≤ 0.01, ^***^p ≤ 0.001.

To confirm the existence of back-splicing for the six candidate circRNAs, we used divergent PCR primers that do not overlap the back-spliced exon-exon junctions to amplify specifically each candidate circRNA. Additional convergent PCR primers were used to corroborate the occurrence of transcription within these genomic loci. Using cDNA from the normal colon mucosa sample, we detected amplification with both convergent and divergent primers, except for TUSC3 circ104558 where only convergent PCR amplification was present (Figure 2A). The presence of head-to-tail splicing of TUSC3 circ104557, ATRNL1 circ100686, POMT1 circ104948, POMT1 circ104949 and SAMD4A circ101356 was validated by Sanger sequencing the PCR fragments amplified by divergent amplification primers (Figure 2B). The microarray observed association between promoter CpG island hypermethylation of the host gene and both linear and circRNA low levels was confirmed by quantitative real-time PCR. TUSC3, ATRNL1, POMT1, and SAMD4A methylated HCT-116 cells showed downregulation of their linear and corresponding circRNAs (TUSC3 circ104557, ATRNL1 circ100686, POMT1 circ104948, POMT1 circ104949 and SAMD4A circ101356), whereas expression of both types of RNA transcript was restored in DKO cells (Figure 2C). The expression levels of both circular and linear transcripts normalized independently with three housekeeping genes (GAPDH/HPRT1/TBP) are shown in Supplementary Figure 2. Interestingly for the ten host genes that did not show DNA methylation differences between HCT-116 and DKO cells, the reactivation of the circular RNA in these last cells was not always significantly associated with a concordant reactivation of its corresponding linear RNA (Supplementary Figure 3), suggesting the existence in these cases of alternatives mechanisms of RNA regulation.

**Figure 3:**
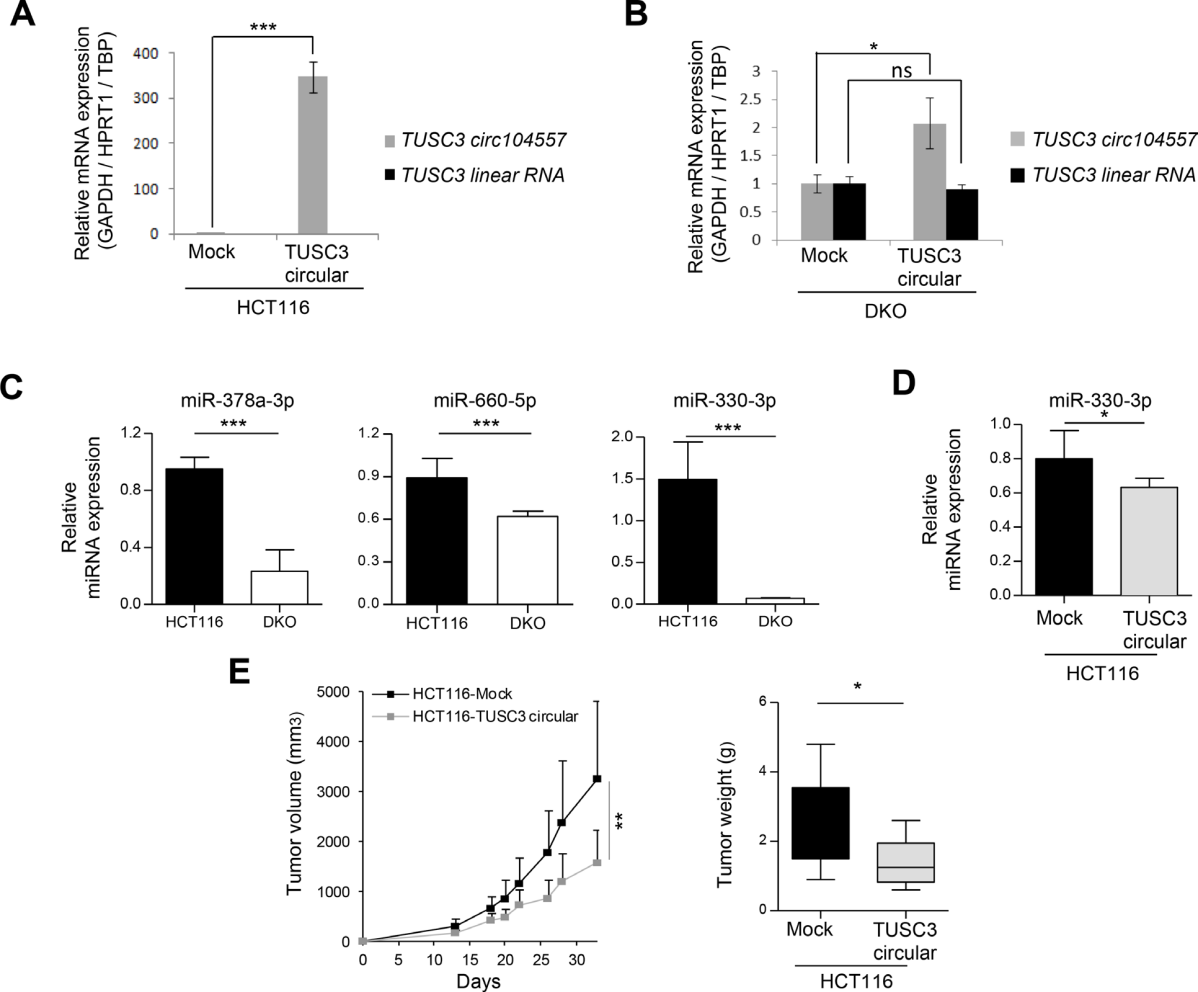
circRNA effects on linear transcripts, miRNAs and tumor growth (**A**) Upon efficient transduction of TUSC3 circ104557 in HCT-116 cells (harboring a methylated CpG island), the TUSC3 linear RNA levels did not change: it was not detected in any of the conditions tested. (**B**) TUSC3 circ104557 transduction in DKO cells (harboring an unmethylated CpG island) did not affect the levels of TUSC3 linear RNA. RNA levels were determined using circular or linear specific qRT-PCR primers. The lentiviral transduction of the empty vector (Mock condition) was used as a control. Experiments were performed in technical triplicates. (**C**) Expression of candidate miRNAs putatively targeted by ATRNL1 circ100686 (miR-378a-3p), SAMD4A circ101356 (miR-660-5p and miR-330-3p) and TUSC3 circ104557 (miR-330-3p) was significantly downregulated in DKO cells, evaluated in three biological replicates by real-time quantitative PCR using TaqMan Advanced MicroRNA Assays. Expression levels were normalized using hsa-miR-345-5p, hsa-miR-191-5p and hsa-miR-423-3p advanced Control miRNA Assays. (**D**) Using the same strategy, expression of miR-330-3p, putatively targeted by TUSC3 circ104557, was also assessed in the gain-of-function cellular model. A significant downregulation of miR-330-3p was detected upon TUSC3 circ104557 transduction in HCT116 cells. (**E**) HCT116-Mock and HCT116-TUSC3 circular cells were injected in the left or right flank of 10 mice, respectively. Tumor volume measured over time (left panel) and tumor weight upon sacrifice (right panel) are shown. Tumor growth was significantly reduced upon TUSC3 circular ectopic expression. ns, nonsignificant; ^*^p ≤ 0.05; ^**^p ≤ 0.01; ^***^p ≤ 0.001, using Student’s *t*-test. Error bars show means ± s.d.

At the mechanistic level, circRNAs seem to possess different functionalities [16, 17, 20]. One possibility is that they regulate linear mRNA gene expression of the host gene [16, 17, 20]. In this regard, the cirRNAs ci-ankrd52, ElciEIF3j and circFoxo3 upregulate the expression of their corresponding host genes ANKRD52 [30], EIF3J [31] and Foxo 3 [22]. To assess the potential impact of the circRNAs studied herein upon the linear RNA derived from the host gene, we focused on TUSC3 circ104557 due to the well-recognized feature of its parental gene TUSC3 as a tumor suppressor gene [32]. To explore its potential activity, we transduced a TUSC3 circ104557 lentiviral expression construct and the empty vector in the colorectal cancer cell lines studied. Upon efficient restoration of TUSC3 circ104557 expression in HCT-116 cells (that exhibit DNA methylation-associated silencing of both the linear and the circRNA), we did not observe any reactivation of linear mRNA expression of TUSC3 (Figure 3A). Importantly, the overexpression of TUSC3 circ104557 in DKO cells (that show an unmethylated status and expression of both linear and circRNA) did not change TUSC3 linear mRNA levels (Figure 3B). The expression levels of both circular and linear TUSC3 transcript normalized independently with three housekeeping genes (GAPDH/HPRT1/TBP) are shown in Supplementary Figure 4. Thus, the circRNA studied does not seem to function through the regulation of the linear RNA levels of its host gene.

Another possible activity of circRNAs is a role as miRNA sponges or decoys to regulate the levels of these molecules [16, 17]. To explore this function, we first used the colorectal cancer cell line HCT-116 (with DNA methylation associated silencing of the studied circRNAs) and the DKO cells (with demethylation-associated reactivation of circRNAs due to the genetic disruption of DNMTs) and studied microRNAs that show base pair complementarity with the identified circRNAs. For HCT-116, we found that the epigenetic silencing of ATRNL1 circ100686, SAMD4A circ101356 and TUSC3 circ104557 is associated with expression of their complementary miRNAs: hsa-miR-378a-3p (ATRNL1 circ100686), hsa-miR-330-3p and hsa-miR-660-5p (SAMD4A circ101356) and hsa-miR-330-3p (TUSC3 circ104557), respectively. In contrast, the reactivation of these circRNAs in DKO was associated with a down-regulation of the described target miRNAs (Figure 3C). In a second experiment, we used the empty-vector (mock) transduced HCT-116 cells in comparison with the TUSC3 circ104557 transduced HCT-116 cell line. We observed that the efficient restoration of TUSC3 circ104557 in HCT-116 downregulates the expression levels of its candidate miRNA target, hsa-miR-330-3p (Figure 3D). Thus, these data suppot a role of the studied circRNAs as miRNAs decoys.

Once we demonstrated the presence of cancer-specific promoter CpG island hypermethylation silencing of the described circRNAs and their proposed activity as miRNA sponges, we examined its contribution to the tumorigenic phenotype. We tested the ability of TUSC3 circ104557-transduced HCT-116 cells to form subcutaneous tumors in nude mice compared with empty vector-transduced cells. We found that empty vector cells formed tumors with a greater weight and volume, but TUSC3 circ104557-transduced HCT-116 cells showed much lower tumorigenicity (Figure 3E). TUSC3 circ104557 ectopic expression in tumors generated upon injection of HCT116-circTUSC3 cells in mouse flanks was confirmed by Real-time qPCR (Supplementary Figure 5). Thus, TUSC3 circ104557 acts in our model as an inhibitor of tumor growth.

### Aberrant DNA methylation profiles of the circular and linear RNA loci identified are a common event in human tumorigenesis

**Figure 4:**
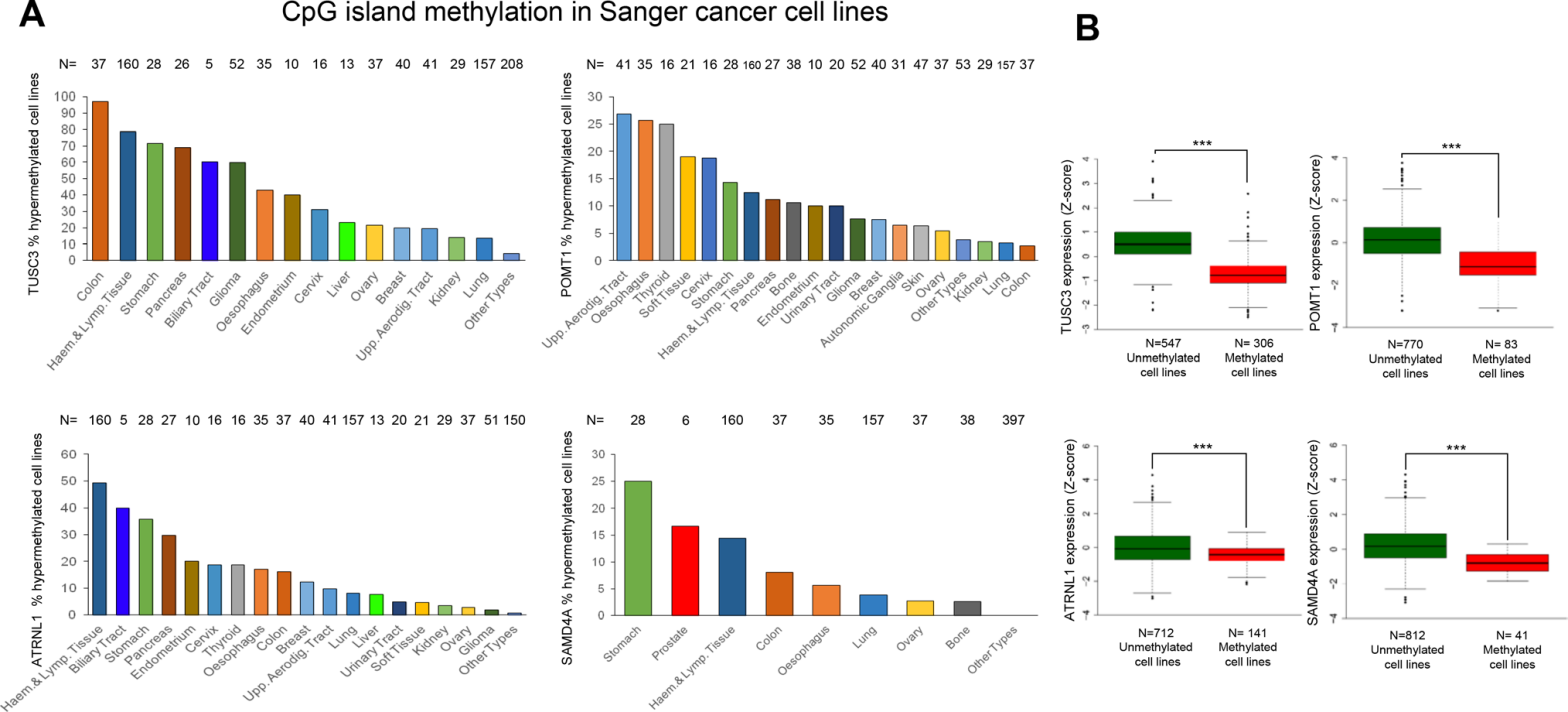
Hypermethylation profiles for the circRNA host genes in cancer cell lines and their association with transcript down-regulation (**A**) Percentage of TUSC3, ATRNL1, POMT1 and SAMD4A promoter CpG island hypermethylated samples in the Sanger panel of cancer cell lines by tumor type. “Haem.”, haematological; “Lymp”, lymphoid; “Aerodig.”, aerodigestive; “Upp.”, upper. (**B**) Promoter CpG island hypermethylation of the genes studied is significantly associated with downregulation of the corresponding transcript in the Sanger set of cancer cell lines. The box plots display the distribution of the expression values with the central solid line representing the median and the limits of the box, the upper and lower quartiles. The whiskers represent the minimum and maximum values excluding outliers (<1.5 × the interquartile range).

The existence of tumor-specific promoter CpG island hypermethylation of the identified circRNA host genes was not a feature observed exclusively in the colorectal cancer cell line HCT-116. Data mining of a collection of 1,001 human cancer cell lines from the Sanger Institute, studied by the same DNA methylation microarray platform herein used, [33] confirmed the existence of hypermethylation of TUSC3, ATRNL1, POMT1 or SAMD4A in a significant proportion of these transformed cells across different tumor types (Figure 4A). The detailed analyses of the 5′-end CpG island methylation status of TUSC3, ATRNL1, POMT1 and SAMD4A in a set of 50 additional human colorectal cancer cell lines beyond HCT-116, including their microsatellite instability status, is shown in Supplementary Figure 6. In addition to colorectal cancer cell lines, the tumor type of the HCT-116 cells used originally, each gene showed a characteristic profile of promoter hypermethylation. TUSC3 and ATRNL1 were predominantly methylated in cancer cell lines derived from the digestive system, such as stomach, pancreas and biliary tract, although their hypermethylation was also very common in hematological malignancies. POMT1 was found more frequently methylated in upper aerodigestive tract-derived tumors and esophagus, thyroid and soft tissue-originated cancer cell lines. SAMD4 showed a mixed pattern in comparison to the other three genes with hypermethylation in digestive system-derived cells (stomach and colon), but also leukemia/lymphoma and esophagus. Interestingly, SAMD4 was the only gene displaying promoter hypermethylation in prostate-derived cancer cell lines. Most importantly, because expression microarray data for all these cell lines are available [33], we proceeded to compare the RNA levels and the DNA methylation status for the four identified circRNA host genes. We found that ATRNL1, POMT1, SAMD4A or TUSC3 promoter hypermethylation in the cell lines studied was associated with downregulation of their corresponding transcripts (Figure 4B).

**Figure 5:**
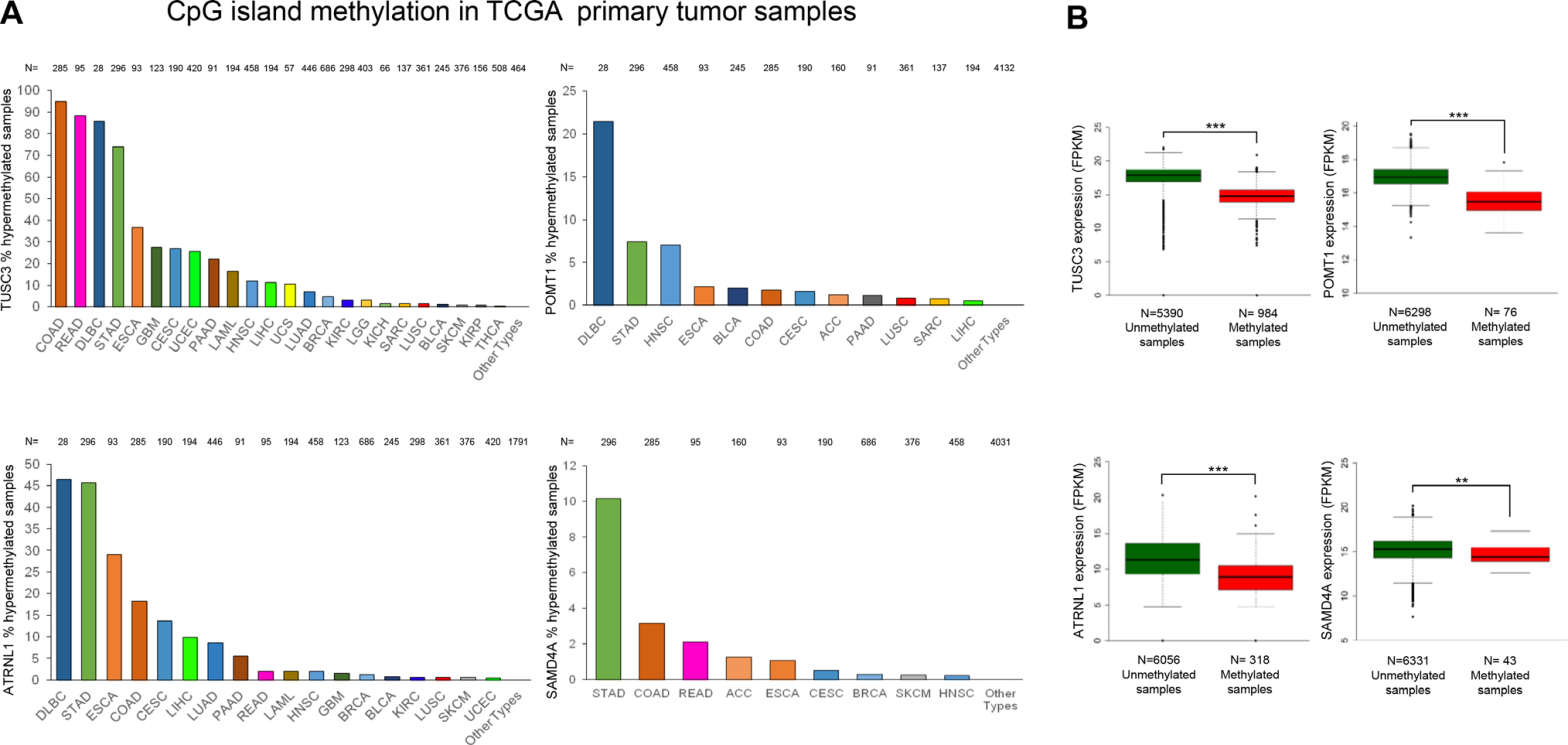
Hypermethylation profiles for the circRNA host genes in primary tumors and their association with transcript down-regulation (**A**) Percentage of TUSC3, ATRNL1, POMT1 and SAMD4A promoter CpG island hypermethylated cases in the TCGA panel of primary tumors by tissue type. Barr codes: Adrenocortical carcinoma (ACC), Bladder urothelial carcinoma (BLCA), Breast invasive carcinoma (BRCA), Cervical and endocervical cancers (CESC), Cholangiocarcinoma (CHOL), Colon adenocarcinoma (COAD), Lymphoid Neoplasm Diffuse Large B-cell Lymphoma (DLBC), Esophageal carcinoma (ESCA), FFPE Pilot Phase II (FPPP), Glioblastoma multiforme (GBM), Glioma (GBM/LGG), Head and Neck squamous cell carcinoma (HNSC), Kidney Chromophobe (KICH), Kidney renal clear cell carcinoma (KIRC), Kidney renal papillary cell carcinoma (KIRP), Acute Myeloid Leukemia (LAML), Brain Lower Grade Glioma (LGG), Liver hepatocellular carcinoma (LIHC), Lung adenocarcinoma (LUAD), Lung squamous cell carcinoma (LUSC), Mesothelioma (MESO), Ovarian serous cystadenocarcinoma (OV), Pancreatic adenocarcinoma (PAAD), Pheochromocytoma and Paraganglioma (PCPG), Prostate adenocarcinoma (PRAD), Rectum adenocarcinoma (READ), Sarcoma (SARC), Skin Cutaneous Melanoma (SKCM), Stomach adenocarcinoma (STAD), Testicular Germ Cell Tumors (TGCT), Thyroid carcinoma (THCA), Thymoma (THYM), Uterine Corpus Endometrial Carcinoma (UCEC), Uterine Carcinosarcoma (UCS) and Uveal Melanoma (UVM). (**B**) Promoter CpG island hypermethylation of the genes studied is significantly associated with downregulation of the corresponding transcript in the TCGA panel of primary tumors. The box plots display the distribution of the expression values with the central solid line representing the median and the limits of the box, the upper and lower quartiles. The whiskers represent the minimum and maximum values excluding outliers (<1.5 × the interquartile range).

The occurrence of TUSC3, ATRNL1, POMT1 or SAMD4A promoter CpG island hypermethylation was not a restricted *in vitro* phenomenon of long-cultured cancer cell lines. Data mining of the collection of primary human tumors from The Cancer Genome Atlas (TCGA) project (https://cancergenome.nih.gov/), assessed by the same DNA methylation microarray used herein [29], showed the existence of 5′-end CpG island hypermethylation of these circRNA host genes in a wide spectrum of malignancies that resembled the cancer cell line data (Figure 5A). In this regard, 5′-end CpG island methylation of TUSC3, ATRNL1, POMT1 and SAMD4A was frequently observed in Colon Adenocarcinoma (COAD), Rectum Adenocarcinoma (READ), Stomach Adenocarcinoma (STAD), Lymphoid Neoplasm Diffuse Large B-cell Lymphoma (DLBC), Esophageal Carcinoma (ESCA), and Head-Neck Squamous Cell Carcinoma (HNSC). As we also did for the cancer cell lines, we were able to compare the TCGA described profiles for the four genes with the available RNA-sequencing data from the same primary tumor samples (https://cancergenome.nih.gov/). We observed that TUSC3, ATRNL1, POMT1 and SAMD4A 5′-end CpG island methylation was again associated with downregulation of their corresponding transcripts (Figure 5B), further tightening the link between the epigenetic mark and transcriptional inactivation.

## DISCUSSION

Promoter CpG island hypermethylation-associated silencing of tumor suppressor genes is a hallmark of human cancer [34]. The coding genes affected by this epigenetic lesion cover all the molecular pathways related to cancer biology, such as DNA repair (BRCA1, MHL1 and MGMT), cell cycle (p16^INK4a^ and p15^INK4b^), apoptosis (DAPK and TMS1) or cell adherence (E-cadherin and cadherin-11) [34]. In the last decade, the increasingly recognized role of noncoding RNAs in carcinogenesis has also created a great interest in the area, and there are now many examples of different noncoding RNA subclasses that undergo DNA methylation-associated expression loss in tumorigenesis [34]. However, there are no reports studying the possible existence of aberrant DNA methylation events in the genomic loci that originate an emerging subtype of ncRNAs, the circRNAs. In this regard, it is important to fill this gap in our knowledge because circRNAs show drastic changes in their expression levels in human cancer, some of them have been shown to exert cellular activities as *bona fide* oncogenes or tumor suppressor genes, and they are starting to prove to be promising cancer biomarkers due their long half-life related to their resistance to RNA exonucleases [16, 17, 20].

Herein, using a combination of epigenomic and transcriptomic platforms, we have shown that certain circRNA genomic loci undergo cancer-specific DNA hypermethylation events. The gain of DNA methylation occurs at the CpG islands located at the 5′-end regulatory regions of the genes hosting the circRNAs. The promoter hypermethylation described silences both the linear RNA isoform of the parental gene and the circular RNA arising from back-splicing of the exons of the linear transcript. Thus, this is an example of how one epigenetic lesion in cancer can cause two molecular “hits”. A similar scenario has been described for bidirectional promoter-associated CpG islands that silence two genes simultaneously, as has been shown for WNT9A/CD558500, CTDSPL/BC040563, and KCNK15/BF195580 [35].

Of the four identified genes hosting circRNAs that undergo hypermethylation-associated downregulation, only TUSC3 had been previously reported to be epigenetically inactivated in human cancer [32]. TUSC3 epigenetic loss has been found in colorectal cancer, the same tumor type in which we initiated our screening, and it is associated with adverse prognosis [36]. Related to its candidate tumor suppressor function, the TUSC3 protein localizes to the endoplasmic reticulum and is involved in protein glycosylation, enabling it to inhibit cell proliferation and promote both apoptosis and autophagy [37]. For the other three newly identified hypermethylated genes, POMT1 is involved in protein glycosylation (as also described above for TUSC3) and has been found to be related to the development of hematological malignancies [38], a tumor type also enriched in our cell line and TCGA methylation screening; ATRNL1 regulates energy homoeostasis and has been found inserted in the EML4-ALK fusion gene [39]; and SAMD4A is an RNA-binding protein linked to drug resistance in cancer cells [40]. Our results pave the way to study the extent to which the putative tumor suppressor functions of these genes are associated with their linear RNA form, circular RNA form, or both.

In summary, our findings highlight that circRNAs undergo loss of expression in human cancer associated with hypermethylation of the 5′-end regulatory CpG island of their corresponding host genes. The promoter hypermethylation described is linked to the loss of both the linear and the circular RNA molecules. We have not observed a direct effect of the circRNA in the associated linear RNA level, however, it affects the levels of target miRNAs. Most importantly, the epigenetic events described at the circRNA genome loci are commonly observed among different types of human malignancies and their roles in the origin and progression of these tumors warrant further research.

## MATERIALS AND METHODS

### Human cancer cell lines

The human colon cancer cell line HCT-116 and its isogenic variant DKO were generous gifts from Dr Bert Vogelstein (Johns Hopkins Kimmel Comprehensive Cancer Center, Baltimore, MD, USA). All cell lines were cultured in Dulbecco’s Modified Eagle’s Medium (DMEM) containing 10% FBS, penicillin and streptomycin, at 37° C in a humidified atmosphere of 5% (v/v) carbon dioxide. Cells were authenticated by STR profiling and tested for the absence of mycoplasma.

### circRNA expression microarray

Total RNA was extracted using Trizol (Thermo Fisher Scientific) and three RNA biological replicates from each sample (HCT-116, DKO and normal colon mucosa) were quantified using the NanoDrop ND-1000 and RNA were assessed by electrophoresis on a denaturing agarose gel. Sample labeling and array hybridization were performed according to the manufacturer’s protocol (Arraystar Inc.). Briefly, total RNA samples were treated with RNase R (Epicentre, Inc.) to remove linear RNAs. Then, each sample was amplified and transcribed into fluorescent cRNA utilizing a random priming method (Arraystar Super RNA Labeling Kit; Arraystar). The labeled cRNAs were purified by RNeasy Mini Kit (Qiagen). Both the concentration and specific activity of the labeled cRNAs (pmol Cy3/μg cRNA) were measured by NanoDrop ND-1000. 1 μg of each labeled cRNA was fragmented by adding 5 μl 10 × Blocking Agent and 1 μl of 25 × Fragmentation Buffer, then the mixture was heated at 60° C for 30 min. Finally, 25 μl 2 × Hybridization Buffer was added to dilute the labeled cRNA. 50 μl of hybridization solution was dispensed into the gasket slide and assembled on the circRNA expression microarray slide. The labeled cRNAs were hybridized onto the Arraystar Human circRNA Array (8 × 15 K, Arraystar). The slides were incubated for 17 hours at 65°C in an Agilent Hybridization Oven. The hybridized arrays were washed, fixed and scanned using the Agilent Scanner G2505C. Agilent Feature Extraction software (version 11.0.1.1) was used to analyze acquired array images. Quantile normalization and subsequent data processing were performed using the R software package. Differentially expressed circRNAs with statistical significance between two groups were identified through Volcano Plot filtering. Differentially expressed circRNAs between two samples were identified through fold change filtering.

### Genome-wide DNA methylation analysis

Genome-wide DNA methylation analysis was performed with Illumina’s 450 K DNA methylation microarray (InfiniumHumanMethylation450 BeadChip) as previously described (29). After sodium bisulfite treatment with an EZ DNA Methylation-Gold kit (Zymo Research), we hybridized in the microarray DNA from the human colon cancer cell lines HCT-116 and DKO and from normal colon mucosa. A three-step normalization procedure was performed using the lumi package, available from Bioconductor, within the R statistical environment. This consisted of color-bias adjustment, background-level adjustment, and quantile normalization across arrays. The methylation level (β-value) of CpG sites was calculated as the ratio of methylated signal to the sum of methylated and unmethylated signals plus 100. Probes were considered to be in a promoter CpG island if they were located within a CpG island (UCSC database) and < 1,500 bp away from a transcription start site.

### Back-splicing of the candidate circular RNAs

Retro-transcribed cDNA corresponding to 50 ng of total RNA was amplified by PCR to confirm the expression of the candidate circRNAs in normal colon (Ambion, Thermo Fisher Scientific, Cat. # AM7986). Divergent PCRs using primers that do not overlap the back-spliced exon-exon junction were used to specifically amplify each candidate circular RNA and to validate the existence of back-splicing. Additional convergent PCRs were used to corroborate the occurrence of transcription within these genomic loci. Importantly, the latter PCR amplify both linear and circular transcripts (one or more). Since circRNAs encoded within POMT1 host gene shared a common sequence, convergent primers were designed on that particular region. On the other hand, although there are two candidate circular RNAs encoded within the TUSC3 gene, they do not share a common sequence and specific convergent primers were designed accordingly. Since SAMD4A circ101356 is a monoexonic circular RNA, a RT- control was used in the convergent amplification, validating the absence of gDNA contamination. Sanger Sequencing (direct or through pGEM^®^-T vector cloning, Promega) was used to sequence the head-to-tail junctions present in the fragments amplified by divergent PCR amplification. The occurrence of back-splicing was confirmed by chromatogram analysis.

### Quantitative PCR

Total RNA samples of the colon cancer cell lines HCT116 and DKO were purified using the Maxwell^®^ RSC miRNA Tissue Kit (Cat. #AS1460, Promega) in the automated Maxwell^®^ RSC Instrument (Promega), according to the manufacturer’s instructions. Human colon total RNA was obtained from Ambion, Thermo Fisher Scientific (Cat. # AM7986). Total RNAs were retro-transcribed using the ThermoScript™ RT–PCR System (Thermo Fisher Scientific), or GoScript Reverse Transcriptase (Promega), with random primer hexamers. Gene expression was determined by quantitative real-time PCR using SYBR Green (Thermo Fisher Scientific) according to the manufacturer’s recommendations. Target gene expression levels were normalized to three housekeeping genes (GAPDH, HPRT1 and TBP). Some of the primers used to detect circular RNAs by divergent PCR were re-designed to overlap the back-spliced exon-exon junction in order to avoid unspecific RT-qPCR products. Primers designed to amplify linear transcripts do not overlap annotated circRNAs according to circBase [41]. Primer sequences are shown in Supplementary Table 1. Quantification of miRNA expression was performed using TaqMan Advanced MicroRNA Assays (ThermoFisher, Cat. No. A25576), according to the manufacturer’s instructions. In brief, TaqMan™ Advanced miRNA cDNA Synthesis Kit (ThermoFisher, Cat. No. A28007) was used for retrotranscription. Next, RT-qPCR was performed using TaqMan Fast Advanced Master Mix (ThermoFisher, Cat. No. 4444557) and specific TaqMan assays. Advanced Control miRNA Assays hsa-miR-345-5p, hsa-miR-191-5p and hsa-miR-423-3p (ThermoFisher, Cat. No. A25576) were used as endogenous controls. TaqMan assays IDs are listed in Supplementary Table 1.

### Validation of DNMT status in KO cells

In order to validate DNMT1 and DNMT3B knockout in DKO cells, semi-quantitative PCRs (30 cycles) were performed using the primers listed in Supplementary Table 1. GAPDH was used as endogenous control. Knockout of DNMT1 and DNMT3B in DKO cells was also confirmed by western blot, using the following antibodies: Anti-DNMT1 from Boster, Cat No. Cl1105; and Anti-DNMT3B from Sigma, Cat. No. HPA001595. In both cases, membranes were incubated overnight in 1:500 diluted antibodies in 5% Milk PBS-T. LaminB1 (Abcam, ab16048, 1:5000) was used as endogenous control.

### Transduction

For the ectopic overexpression of TUSCS3_circ104557, the cDNA corresponding to the circularized exons was amplified, adding artificial flanking sequences to promote circularization, as previously done [42]. Two sequential PCRs were performed to amplify the sequence comprising the circularized exons, the flanking regions and EcoRI or NotI restriction sites. This sequence was cloned into pLVX-ZsGreen1 plasmid from Clontech. 10 µg of each plasmid were mixed with 7.5 µg of psPAX2 and 2.5 µg of pMD2.G plasmids in 1ml of JetPRIME buffer and 50 µl of JetPRIME (Polyplus-transfection S.A.). Cloning oligonucleotides are shown in Supplementary Table 1. After 10 min of RT incubation, the transfection mix was added drop-wise to a 10-cm dish containing 10 ml of DMEM and Lenti-X 293T cells (Clontech) at 80% confluence to produce lentivirus. After 72 h, the supernatant with high-titer lentiviral particles was recovered and 0.45-µm filtered. HCT-116 or DKO cells were incubated with 1.5 ml of concentrated viral supernatant supplemented with 10 μg/ml of polybrene (Santa Cruz Bitechnology) in six-well plates. A high transduction efficiency for all conditions was confirmed at the microscope by the presence of green fluorescence (ZsGreen1).

### Mouse xenografts

Five-week-old male athymic nu/nu mice (Charles River, Wilmington, MA, USA), housed under specific pathogen-free conditions, were used in this study. In order to assess tumor growth, 3.5 × 10^6^ cells were subcutaneously injected in each flank of the mouse. The left flank was used for HCT116 control cells (mock) and the right for the circTUSC3 stably transduced HCT116 cells. From day 13 after implantation, tumor growth was monitored every 3–5 days and the tumor width (W) and length (L) were measured. Tumor volume was estimated according to the formula V = π/6 × L × W^2^. Mice were sacrificed 33 days after injection, and tumors were then excised and weighed. Then, tumor tissues were homogenized using liquid nitrogen, and RNA was extracted using the Maxwell^®^ RSC miRNA Tissue Kit (Cat. #AS1460, Promega) in the automated Maxwell^®^ RSC Instrument (Promega), according to the manufacturer’s instructions. After retrotranscription, circTUSC3 expression was quantified by real time PCR using the primers listed in Supplementary Table 1, as described above in Quantitative PCR section.

### Statistical analysis

The associations between variables were assessed by χ^2^ tests, Fisher’s exact test, Welch’s *t*-test, Wilcoxon paired test or Spearman correlation whenever indicated. *P* values less than 0.05 were considered statistically significant. All statistical tests were two-sided. Methylation and expression values for TCGA primary tumor samples were obtained from the NCI’s Genomic Data Commons (GDC). The values corresponding to cancer cell lines were obtained from COSMIC cell line database. Correlations were obtained by calculating Spearman’s rank correlation test and the associated Rho coefficient.

## SUPPLEMENTARY MATERIALS FIGURES AND TABLE




