# Supplementary material for: Circular RNA CpG island hypermethylation-associated silencing in human cancer
Source: Oncotarget. 2018 Jun 26;9(49):29208–19. doi: 10.18632/oncotarget.25673 (PMC6044373; doi:10.18632/oncotarget.25673)
Supplement: Supplementary file 1 [file oncotarget-09-29208-s001.pdf]

Circular RNA CpG island hypermethylation-associated silencing in human cancer

SUPPLEMENTARY MATERIALS

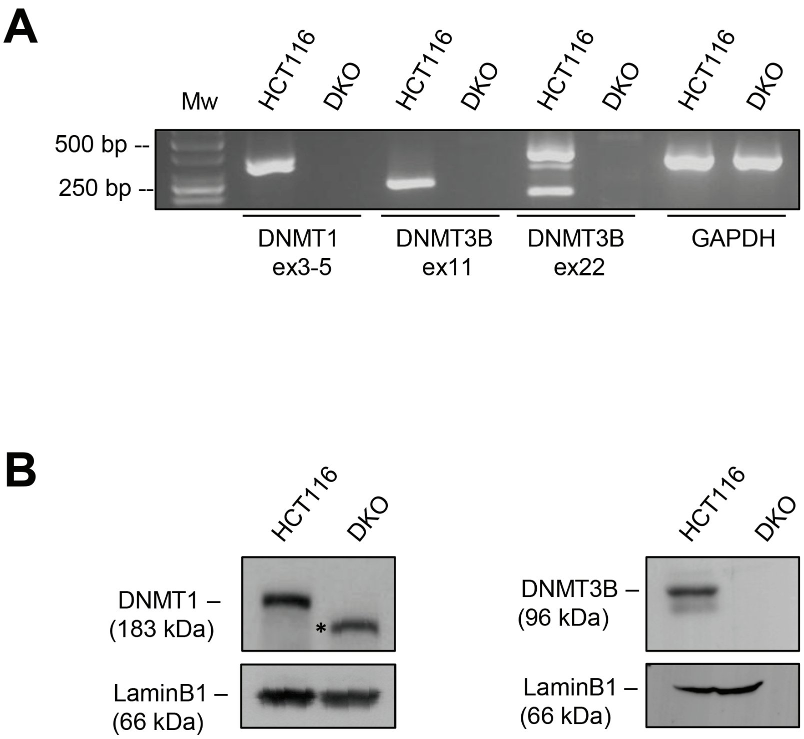

Supplementary Figure 1: Validation of DNMT1 and DNMT3B knockout in DKO cells. Expression of DNMT1 and DNMT3B in HCT116 parental cell line and the DNMT1/DNMT3B doble knockout (DKO) cell line, evaluated by semiquantitative RT-PCR of mRNA (**A**) and by western blot for protein expression (**B**). GAPDH and Lamin B1 were used as endogenous controls in (A) and (B), respectively. The targeting strategy to disrupt DNMT1 resulted in deletion of exons 3, 4, and 5 of wild-type DNMT1, generating a truncated protein, as previously described (Egger G, Jeong S, Escobar SG, Cortez CC, Li TW, Saito Y, Yoo CB, Jones PA, Liang G. Identification of DNMT1 (DNA methyltransferase 1) hypomorphs in somatic knockouts suggests an essential role for DNMT1 in cell survival. Proc Natl Acad Sci U S A. 2006;103:14080–5.)

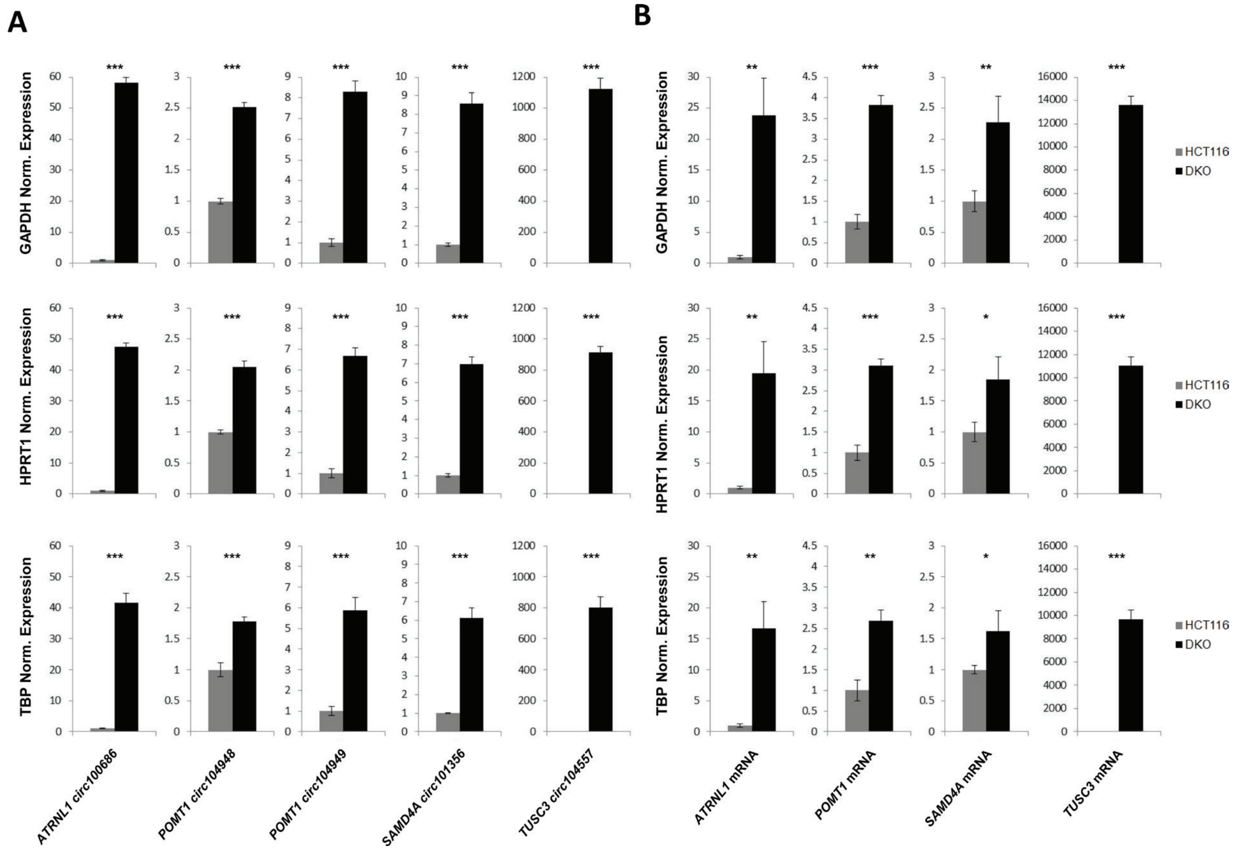

Supplementary Figure 2: Real-time quantitative PCR of both circular (**A**) and linear (**B**) transcripts in HCT-116 and DKO cells. Primers designed to amplify linear transcripts do not overlap annotated circRNAs according to circBase. Expression levels normalized independently with three housekeeping genes (GAPDH/HPRT1/TBP) are shown. Error bars, SD from three biological replicates. $^{*}p \leq 0.05$, $^{**}p \leq 0.01$, $^{***}p \leq 0.001$.

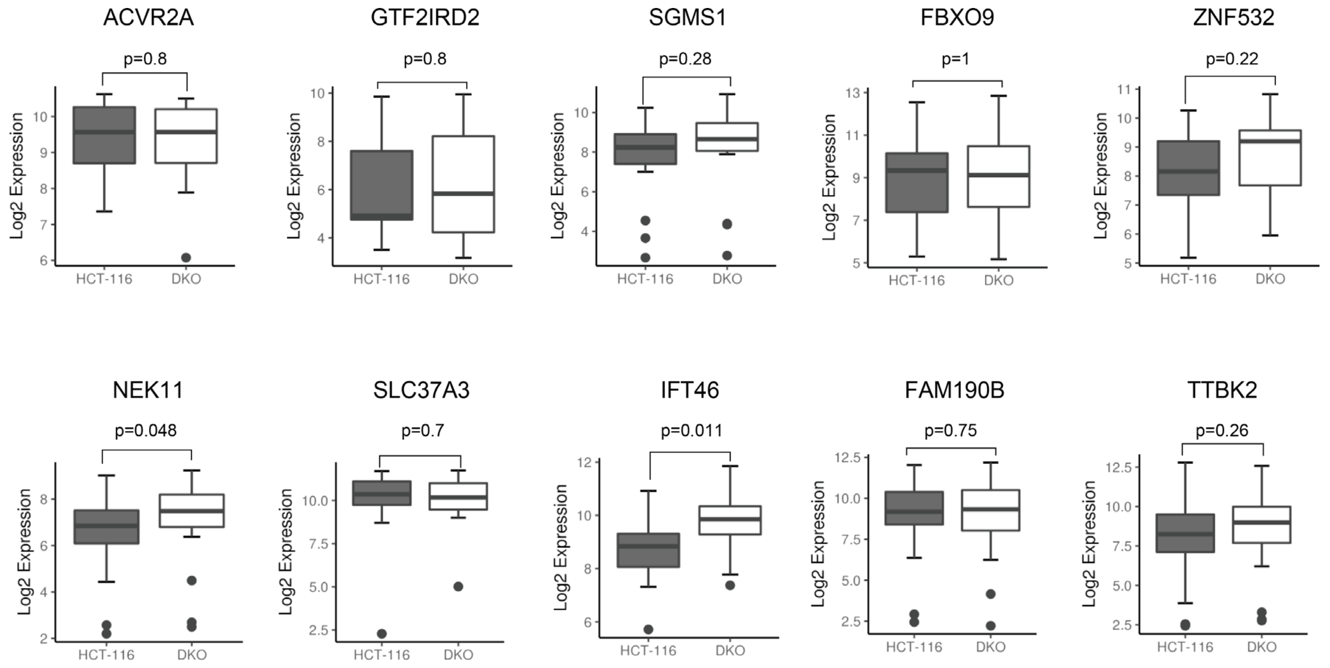

Supplementary Figure 3: Exon gene expression for linear transcripts in HCT-116 and DKO cell lines. The log2 mRNA expression values were calculated from GSE26018 publicly available data. The differential expression significance was calculated by a Wilcoxon test.

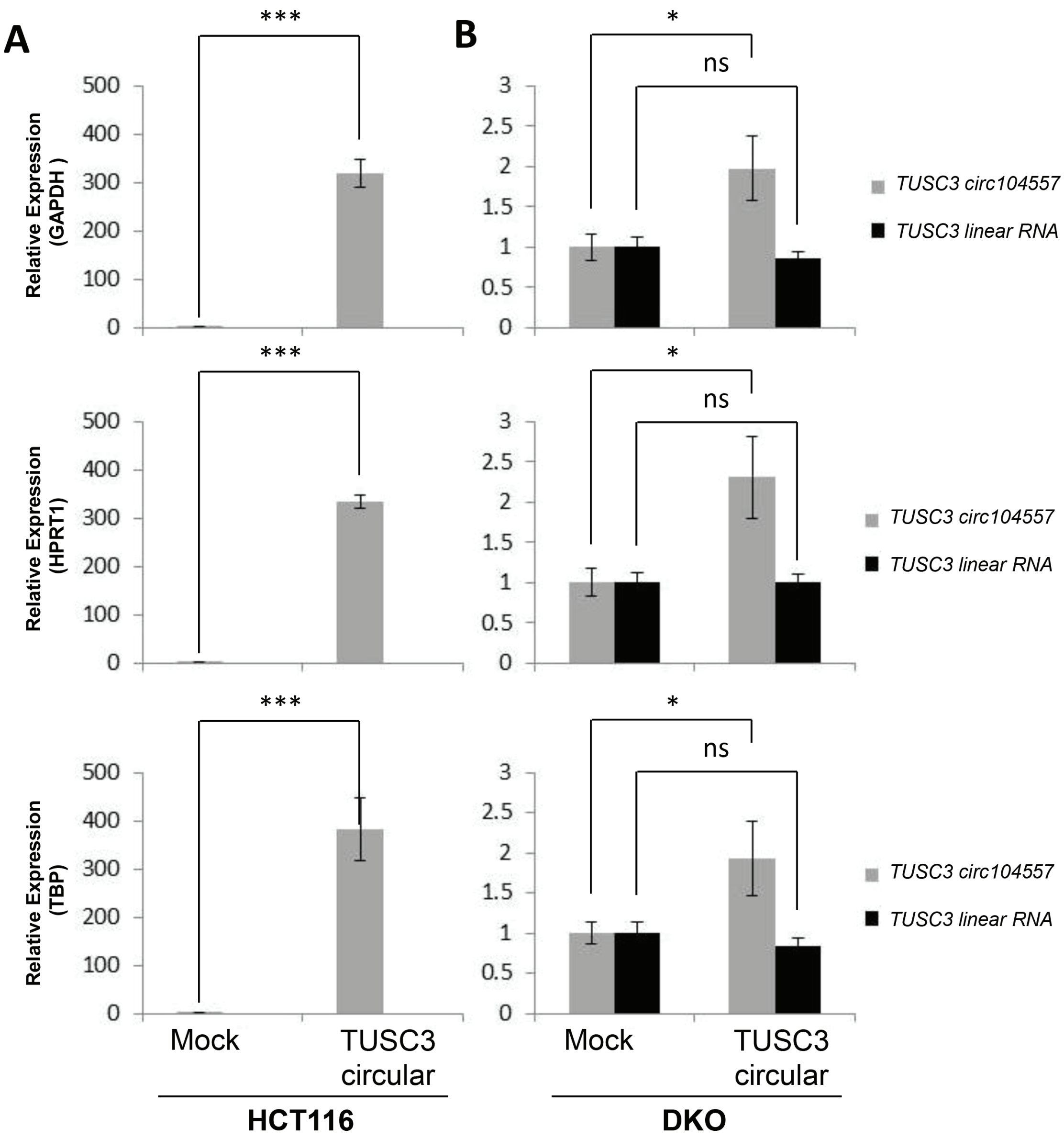

Supplementary Figure 4: TUSC3 circ104557 transduction in HCT-116 (**A**) or DKO (**B**) cells did not affect the levels of TUSC3 linear RNA. Expression levels normalized independently with three housekeeping genes (GAPDH/HPRT1/TBP) are shown. RNA levels were determined using circular or linear specific qRT-PCR primers. The lentiviral transduction of the empty vector (Mock condition) was used as a control. Experiments were performed in technical triplicates. Ns, nonsignificant; $^{*}p \leq 0.05$; $^{***}p \leq 0.001$, using Student's t-test. Error bars show means ± s.d.

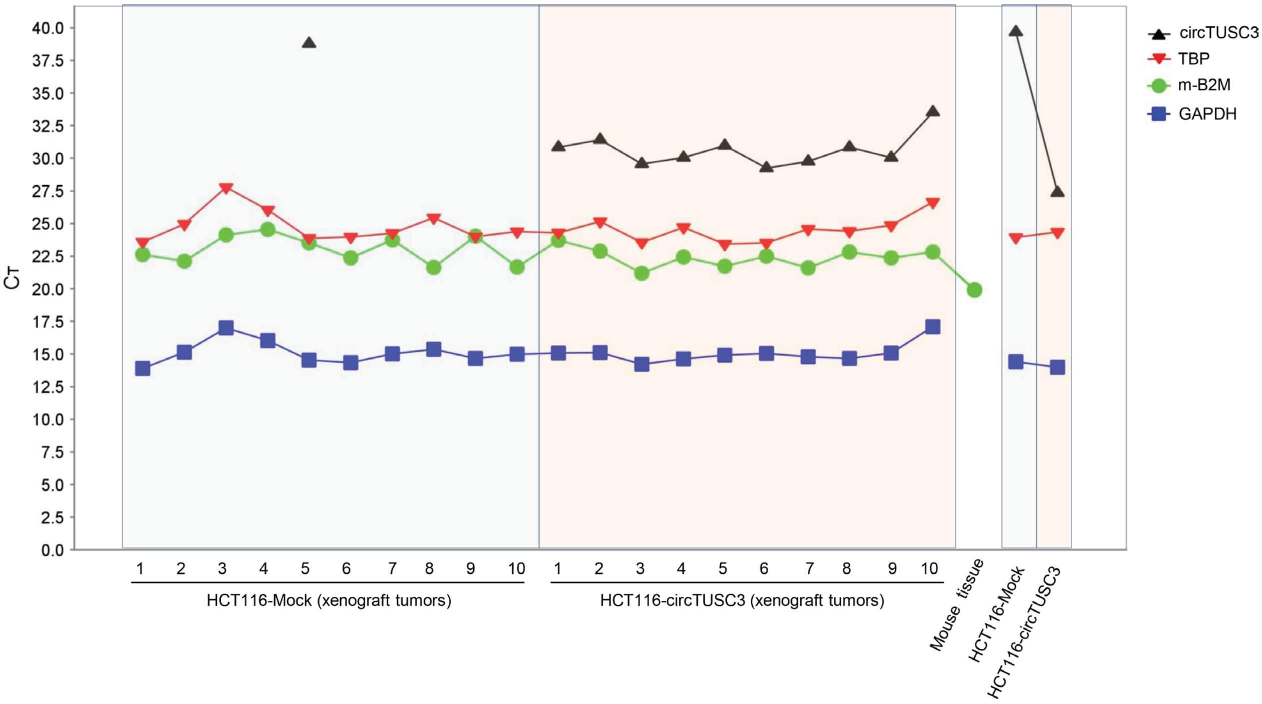

Supplementary Figure 5: circTUSC3 expression in xenograft tumors. circTUSC3 expression in tumors generated upon injection of HCT116-Mock control cells or HCT116-circTUSC3 cells in mouse flanks was confirmed by Real-time qPCR. circTUSC3 levels in cell lines before injection in mice are shown at the right edge. Optimized divergent primers for qPCR (overlaping the back-spliced exon-exon junction) were used to assess circTUSC3 expression. Mouse tissue was used as control. Human TBP, human GAPDH and mouse B2M primers were used as endogenous controls. Ct values are depicted. Most of HCT116-Mock tumors show lack of expression at 40 cycles of qPCR, whereas ectopic expression of circTUSC3 was confirmed in all HCT116-circTUSC3 xenograft tumors.

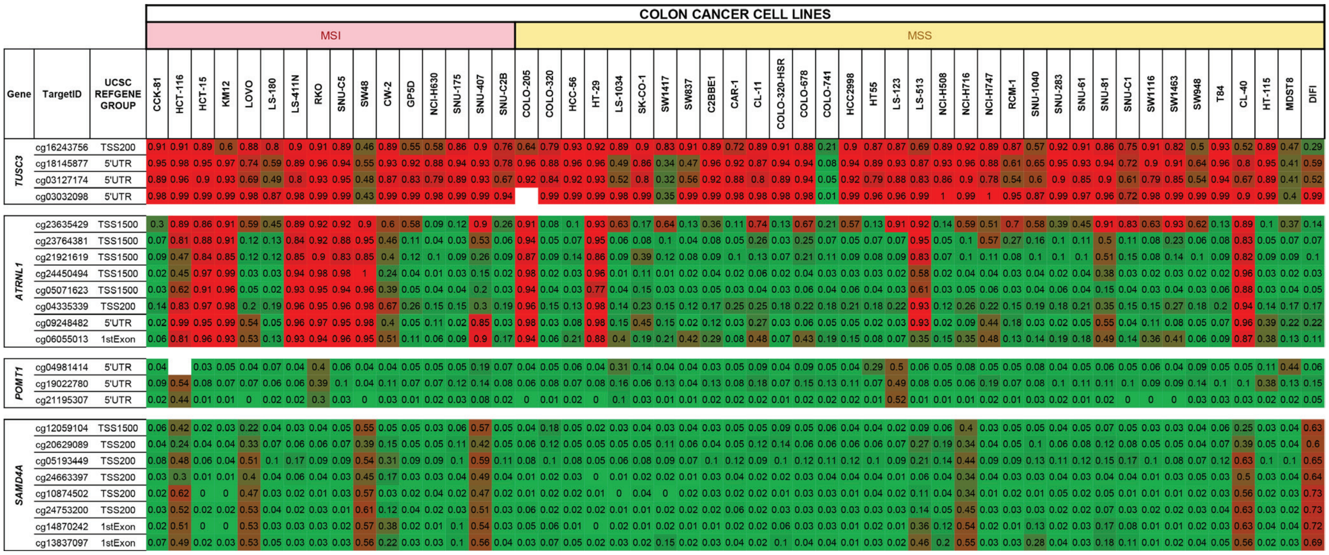

Supplementary Figure 6: Promoter methylation values of CpGs located on genes TUSC3, ATRNL1, POMT1 and SAMD4A for colorectal cancer cell lines obtained from the Sanger Cell Line Project. Colon cancer cell lines are classified according to microsatellite instability (MSI) or microsatellite stable status (MSS). Methylation values are colored according to a continuous color scale (unmethylated-green, methylated-red).

Supplementary Table 1: List of PCR primers, cloning oligonucleotides and TaqMan assays used in the study. See Supplementary_Table_1
