# Supplementary material for: Circular RNA CpG island hypermethylation-associated silencing in human cancer
Source: Oncotarget. 2018 Jun 26;9(49):29208–19. doi: 10.18632/oncotarget.25673 (PMC6044373; doi:10.18632/oncotarget.25673)
Supplement: Supplementary file 2 [file oncotarget-09-29208-s002.docx]

| **Supplementary Table 1: List of PCR primers, cloning oligonucleotides and TaqMan assays used in the study.**  **Convergent Primers** | | | |  |  |  |
| --- | --- | --- | --- | --- | --- | --- |
| **Gene** | **Forward Sequence** | | **Reverse Sequence** |  |  |  |
| ATRNL1 circ100686 | ACTGGTTTCAACATTTTCTATTCAA | | GCTTCACCCTTCCAGTATTT |  |  |  |
| POMT1 circ104948 | TCTTCTTGGATGACAGTGGGC | | TGCTCCAATTCTGTTCCACA |  |  |  |
| POMT1 circ104949 | TCTTCTTGGATGACAGTGGGC | | TGCTCCAATTCTGTTCCACA |  |  |  |
| SAMD4A circ101356 | GAATCACTTGGAGGACCGCA | | CCATTCTCACACCCCATGCT |  |  |  |
| TUSCS3 circ104557 | TGGATTTGCAGCTGAGCAAC | | ACAGGGCCAAAGCAATGGTA |  |  |  |
| TUSCS3 circ104558 | ACACAATGGACAAGTGAGCT | | GGCAAATTATCCGTCTTTTT |  |  |  |
| **Divergent Primers** | | | |  |  |  |
| **Gene** | **Forward Sequence** | | **Reverse Sequence** |  |  |  |
| ATRNL1 circ100686 | AGAAAAATTATGTGTCTGCAATGAT | | AAAGGTGCATATATTGAATCTCCA |  |  |  |
| POMT1 circ104948 | TTTTGTGGAACAGAATTGGAGCA | | GGCCCACTGTCATCCAAGAAG |  |  |  |
| POMT1 circ104949 | ATGGGAGCTGCTCTGTTGAT | | GGCCCACTGTCATCCAAGAAG |  |  |  |
| SAMD4A circ101356 | AATGGGTGGCAGAACTCTCG | | GTCGAGGTTTCCTGGCTTCA |  |  |  |
| TUSC3 circ104557 | GTGGAACCATATCCGTGGAC | | GAAAAACGTCTGTCCCCTCA |  |  |  |
| TUSC3 circ104558 | AAGCAGCAACTTCGAAAGGC | | ATGGTGATAGCGGCATTCAGT |  |  |  |
| **Optimized divergent primers for qPCR (overlap the back-spliced exon-exon junction)** | | | |  |  |  |
| **Gene** | | **Forward Sequence** | **Reverse Sequence** |  |  |  |
| SAMD4A circ101356 | | GGAACCAGCACAAGTACAAGAATC | GTCGAGGTTTCCTGGCTTCA |  |  |  |
| TUSCS3 circ104557 | | GTGGAACCATATCCGTGGAC | TTTGATATTCTTCATTAGCTTGCCAC |  |  |  |
| **Linear qPCR** | | | |  |  |  |
| **Gene** | | **Forward Sequence** | **Reverse Sequence** |  |  |  |
| ATRNL1 mRNA | | ACTGCCAGGGCAGGTTC | TGGATAGCCTTCAATGAGCCA |  |  |  |
| POMT1 mRNA | | TAGTGATCTGGGTTTCGGGC | CCATCAGGAAGAACGGGAGG |  |  |  |
| SAMD4A mRNA | | AGCTGCACGTCCTCGAAC | TCCTTGGATTCCTGTTGCCA |  |  |  |
| TUSC3 mRNA | | TCGGGGGAGGACAGAAGAAA | CGGAAGATTGAGCGTCTGGA |  |  |  |
| **Housekeeping genes** | | | |  |  |  |
| **Gene** | | **Forward Sequence** | **Reverse Sequence** |  |  |  |
| GAPDH mRNA | | TGCACCACCAACTGCTTAGC | GGCATGGACTGTGGTCATGAG |  |  |  |
| HPRT1 mRNA | | TGACACTGGCAAAACAATGCA | GGTCCTTTTCACCAGCAAGCT |  |  |  |
| TBP mRNA | | CACGAACCACGGCACTGATT | TTTTCTTGCTGCCAGTCTGGAC |  |  |  |
| **Additional qPCR primers** | | | |  |  |  |
| **Gene** | | **Forward Sequence** | **Reverse Sequence** |  |  |  |
| DNMT1_ex3-5 | | GCACGAATTTCTGCAAACAG | TGTAATCCTGGGGCTAGGTG |  |  |  |
| DNMT3b_ex11 | | GGAGACTCATTGGAGGACCA | GGCAACATCTGAAGCCATT |  |  |  |
| DNMT3b_ex22 | | ATCTCACGGTTCCTGGAGTG | AAGAGGTGTCGGATGACAGG |  |  |  |
| GAPDH (DNMTs) | | TCTTCTTTTGCGTCGCCAG | AGCCCCAGCCTTCTCCA |  |  |  |
| GAPDH (xenograft) | | CAAGATCATCAGCAATGCCT | AGGGATGATGTTCTGGAGAG |  |  |  |
| TBP (xenograft) | | CACGAACCACGGCACTGATT | TTTTCTTGCTGCCAGTCTGGAC |  |  |  |
| Mouse-B2M | | ACCCGCCTCACATTGAAATCC | CGATCCCAGTAGACGGTCTTG |  |  |  |
|  | |  |  |  |  |  |
| **Cloning Oligos PCR1** | |  |  |  |  |  |
| **Gene** | | **Sequence** | | |  |  |
| F_tag_TUSC3 circ104557 | | GGTACGTACTAATGACTTTTTTTTTATACTTCAGGCAAGCTAATGAAGAATATCAAATACTGGC | | |  |  |
| R_tag_TUSC3 circ104557 | | TGGTCTCTAATTCTTTTCCTTGCTTCTTACCACTTGTCCATTGTGTGGGTTCTTATGAGC | | |  |  |
|  | |  | |  |  |  |
| **Cloning Oligos PCR2** | | | |  | | |
| **Gene** | | **Sequence** | |  |  |  |
| F_ EcoRI_circular | | AAAAAAAAGAATTCAGTAGAGACGGGGTTTCACCATGTTGGCCAGGCTGGTCTTCACTTTTTGTAAAGGTACGTACTAATGACTTTTTTTTTATACTTCAG | |  |  |  |
| R_NotI_circular | | AAAAAAAAGCGGCCGCAGTAGAGACAAGGTTTCACCATGTTGGCCAGGCTGGTCTCTAATTCTTTTCCTTGCTTCTTAC | |  |  |  |
|  | | | |  | | |

| **miRNA expression TaqMan assays** | | | | |  |  |
| --- | --- | --- | --- | --- | --- | --- |
| Assay Name | Assay ID | | | Assay Type |  |  |
|  |  | | | |  |  |
| hsa-miR-20a-3p | | 478317_mir | TaqMan Advanced MicroRNA Assay | | | |
| hsa-miR-211-3p | | 478766_mir | TaqMan Advanced MicroRNA Assay | | | |
| hsa-miR-214-3p | | 477974_mir | TaqMan Advanced MicroRNA Assay | | | |
| hsa-miR-330-3p | | 478030_mir | TaqMan Advanced MicroRNA Assay | | | |
| hsa-miR-378a-3p | | 478349_mir | TaqMan Advanced MicroRNA Assay | | | |
| hsa-miR-448 | | 478105_mir | TaqMan Advanced MicroRNA Assay | | | |
| hsa-miR-558 | | 479044_mir | TaqMan Advanced MicroRNA Assay | | | |
| hsa-miR-660-5p | | 478192_mir | TaqMan Advanced MicroRNA Assay | | | |
| hsa-miR-767-5p | | 479176_mir | TaqMan Advanced MicroRNA Assay | | | |
| hsa-miR-345-5p | | 478366_mir | Advanced Control miRNA Assay | | | |
| hsa-miR-191-5p | | 477952_mir | Advanced Control miRNA Assay | | | |
| hsa-miR-423-3p | | 478327_mir | Advanced Control miRNA Assay | | |  |
